# Monitoring the Secretory Behavior of the Rat Adrenal Medulla by High-Performance Liquid Chromatography-Based Catecholamine Assay from Slice Supernatants

**DOI:** 10.3389/fendo.2017.00248

**Published:** 2017-09-25

**Authors:** Frédéric De Nardi, Claudie Lefort, Dimitri Bréard, Pascal Richomme, Christian Legros, Nathalie C. Guérineau

**Affiliations:** ^1^Mitochondrial and Cardiovascular Pathophysiology – MITOVASC, CNRS UMR6015, INSERM U1083, UBL/Angers University, Angers, France; ^2^EA921, SONAS, SFR QUASAV, UBL/Angers University, Angers, France

**Keywords:** high-performance liquid chromatography, catecholamine release, fluorescence derivatization, stimulus-secretion coupling, acute adrenal slice, medullary tissue, acetylcholine, hexamethonium

## Abstract

Catecholamine (CA) secretion from the adrenal medullary tissue is a key step of the adaptive response triggered by an organism to cope with stress. Whereas molecular and cellular secretory processes have been extensively studied at the single chromaffin cell level, data available for the whole gland level are much scarcer. We tackled this issue in rat by developing an easy to implement experimental strategy combining the adrenal acute slice supernatant collection with a high-performance liquid chromatography-based epinephrine and norepinephrine (NE) assay. This technique affords a convenient method for measuring basal and stimulated CA release from single acute slices, allowing thus to individually address the secretory function of the left and right glands. Our data point that the two glands are equally competent to secrete epinephrine and NE, exhibiting an equivalent epinephrine:NE ratio, both at rest and in response to a cholinergic stimulation. Nicotine is, however, more efficient than acetylcholine to evoke NE release. A pharmacological challenge with hexamethonium, an α3-containing nicotinic acetylcholine receptor antagonist, disclosed that epinephrine- and NE-secreting chromaffin cells distinctly expressed α3 nicotinic receptors, with a dominant contribution in NE cells. As such, beyond the novelty of CA assays from acute slice supernatants, our study contributes at refining the secretory behavior of the rat adrenal medullary tissue, and opens new perspectives for monitoring the release of other hormones and transmitters, especially those involved in the stress response.

## Introduction

Catecholamines (CA) play important physiological roles in human and animal organisms by contributing to maintain body homeostasis. The main source of epinephrine (E), and to a lesser extent of norepinephrine (NE), originates from the neurosecretory chromaffin cell population housed in the adrenal medullary tissue. E and NE are among the first hormones to be released in response to stressful situations, and once delivered into the blood circulation, they exert multiple peripheral actions, in particular on the cardiovascular system, leading to appropriate adjustments of blood pressure and cardiac rhythm, and on the energy metabolism, helping thus the organism to cope with stress (1). The stimulus-secretion coupling underlying adrenal CA release relies on both an incoming synaptic command arising from the splanchnic nerve terminals (2) and a local gap junctional communication between chromaffin cells (3–7).

In basic research, the secretory behavior of the adrenal glands is usually examined through the determination of CA (and/or their metabolites) levels in the systemic blood circulation or in the urines. In clinical medicine, measurements of plasma and urinary CA and/or their metabolite levels can be used to for the diagnosis and the follow-up of several neuroendocrine tumors, such as neuroblastoma (8), paragangliomas (9), pheochromocytomas (10–12), early arterial hypertension (13, 14), and also for evaluating hemodynamic and sympathoadrenal functions in patients in intensive cares (15–17).

Many techniques have been developed for the determination of CA and/or their metabolites [reviewed in Ref. (18–22)]. It includes enzyme immunoassays (23, 24), capillary electrophoresis (25), radioenzymatic assays (26–28), electrochemical methods such as amperometry and cyclic voltammetry (29, 30), but high-performance liquid chromatography (HPLC) remains the more routinely used method enabling the simultaneous assay of several CA (E, NE, and dopamine) and/or their metabolites in biological fluids (20, 31–35).

In rodents, ELISA- or HPLC-based detections were alternatively used to assess CA concentrations, *in vivo* in anesthetized animals, from the adrenal venous blood (6, 36), from microdialyzed adrenal gland (37, 38), in the general blood circulation (39, 40), or even in conscious animals (41). These detection methods are also suitable to assay *ex vivo* CA levels, released from isolated perfused adrenal gland (42) or in collected plasma samples (43, 44). Collectively, these studies provide information on CA released from an individual gland, usually the left one, which is more easily accessible than the right one, or when measuring circulating levels into the plasma or urines, provide a whole secretory profile that merges the left and right gland secretions. When focusing on kinetic parameters of CA secretion, electrochemical methods (amperometry and fast-scan voltammetry) are more appropriate techniques. Indeed, amperometry enables the detection of exocytotic events both in cultured chromaffin cell populations (45), in individual cultured chromaffin cells (46–48), in single chromaffin cells in acute adrenal slices (3, 49, 50), and in perfused cat adrenals (51). Cyclic voltammetry allows the identification of the species detected, as such E and NE can be distinguished (46, 52) and when monitored at a very high scan rate, fast-scan cyclic voltammetry allows CA detection with high spatial and temporal resolution (53).

Even if all techniques cited above to measure CA secretion significantly contributed to improve our knowledge on E and NE release from adrenal chromaffin cells, questions are still unsolved. In particular, whether the two adrenals behave equally or not is to date an open question. It is of a particular interest, keeping in mind that the two glands are distinctively innervated (54), the left adrenal receiving the greatest contribution from T8 and the right gland from T9 (55) and vascularized (56). Also the location of chromaffin cells within the gland, i.e., at the vicinity of the cortical zone versus at the center of the medulla, where the central vein originates from, can impact the secretory behavior. The close and finely tuned interactions between the cortex and the medulla are, with this respect, remarkable (57). These issues remain to be investigated and claim to be addressed in tissue preparations. Indeed, many cellular components involved in the secretory process (cytoskeleton-associated proteins, intracellular organelles, etc.) display differential organization and distribution in isolated cells and in gland pieces, as recently reported in bovine chromaffin tissue (58).

We therefore settled an experimental strategy enabling to simultaneously monitor E and NE secreted from acute adrenal slices derived from either the left or the right gland. We show that the left and right glands are equally competent to secrete E and NE, exhibiting an equivalent E:NE ratio, both at rest and in response to a cholinergic stimulation. In addition, our pharmacological experiments unveil a preferential expression of α3-containing nAChRs in NE cells.

## Materials and Methods

All procedures in this study conformed to the animal welfare guidelines of the European Community and were approved by the French Ministry of Agriculture (authorizations no. 49-2011-18 and 49-247).

### Adrenal Slice Preparation

Acute slices were prepared from 10- to 14-week-old Wistar male rats that have been killed by dioxide carbone inhalation. As previously described (3), after removal, the glands were kept in ice-cold saline for 2 min. A gland was next glued onto an agarose cube and transferred to the stage of a vibratome (DTK-1000, D.S.K., Dosaka EM Co. Ltd., Kyoto, Japan). During the slicing procedure, the gland was immersed into a cold Ringer’s saline containing (in mM): 125 NaCl, 2.5 KCl, 2 CaCl_2_, 1 MgCl_2_, 1.25 NaH_2_PO_4_, 26 NaHCO_3_, 12 glucose and buffered to pH 7.4. Slices of 150 µm thickness were cut with a razor blade and transferred to a storage chamber containing Ringer’s saline, maintained at 37°C under constant carbogenation (95% O_2_/5% CO_2_). Left and right glands were alternatively cut as first. An average of five to six medullary tissue-containing slices was obtained for each gland. Before testing, each slice recovered during 15 min in Ringer’s saline.

### Adrenal Slice Challenge

After 15 min recovery, slices were gently removed from the storage chamber using a cutoff plastic Pasteur pipette, and transferred into an Eppendorf tube. The milieu was removed and replaced by 200 µl pre-warmed and pre-carbogened Ringer’s saline. After 5 min, the supernatant was removed and stored at −20°C. This step referred to basal (B) secretion. Slices were next challenged for 5 min with a test (T) solution containing either 10 µM acetylcholine chloride (ACh, Sigma Aldrich, Saint-Quentin Fallavier, France), 10–100 µM nicotine (tartrate salt, Sigma Aldrich), 200 µM hexamethonium chloride (Sigma Aldrich), or saline as control. The slice supernatant (200 μl) was then collected and stored at −20°C before use. For each slice, results were expressed as the stimulation T/B ratio. Basal and stimulated amounts of E and NE were expressed in picomoles and in picomole per cubic meters of medullary tissue after normalization to the medulla volume.

### Determination of the Medullary Tissue Volume

At the end of the challenge, the medulla upper and lower side surfaces were calculated for each slice. To achieve this, slices were bathed for 2 min in hematoxylin (1 g/l) and rinsed in Ringer’s saline before observation under a binocular microscope. The differential staining intensity between the cortical and the medullary tissues allowed estimating the medulla area. Image processing was performed using ImageJ software. The average between the two surfaces has been calculated and taking into account the 150 μm thickness, a medulla volume was estimated.

### HPLC-Based CA Assay

Benzylamine was used as a pre-column derivatization agent to generate fluorescent benzoxazole derivatives (59). The derivatization reactions were carried out using 20 µl of a standard solution of E and NE or supernatant samples or water (blank) mixed to benzylamine hydrochloride (150 mM in 90% aqueous methanol, Sigma Aldrich), 3-cyclohexylaminopropanesulfonic acid buffer (20 mM, pH 11, Sigma Aldrich) and potassium hexacyanoferrate III (10 mM in 50% aqueous methanol, Sigma Aldrich) (2/1/1, v/v/v). The derivatization procedure was achieved by heating the samples at 50°C for 25 min. A 20 µl fraction of the final reaction mixture was injected into the HPLC system. The fluorescent benzoxazole derivatives were loaded onto a C18 column (Vydac 218TP54, 250 mm × 4.6 mm; 5 µm), and separated on a Waters Separations Module 2695 equipped with a Waters multi-wavelength fluorescence detector 2475 (33). The separation of the benzylamine derivatives was achieved using a mixture of 10 mM acetate buffer (pH 5.5) containing acetonitrile (VWR International MERCK, Fontenay-sous-Bois, France, 65/35, v/v). The flow rate ran at 1 ml/min (isocratic mode during 15 min) and the column temperature was maintained at 23°C. The detection was monitored at an excitation wavelength of 345 nm and an emission wavelength of 480 nm. To determine E and NE concentrations, the areas under the peaks of samples were compared to the peaks of the standards [E (Sigma Aldrich) and NE (VWR International MERCK), bitartrate salts] used as external calibrator. The limits of detection (LOD, with a signal-to-noise ratio of 3) for an injection volume of 20 µl were calculated with the following equation: LOD = 3 × *h*_m_ × *R*, where *h*_m_ corresponds to the maximal baseline noise and *R* to the response factor calculated from the ratio amount/peak height, according to the resolution Oeno 7/2000 (Compendium of International Methods of Analysis).

### Statistical Analysis

Values are presented as the mean ± SEM. GraphPad Prism 7.02 (GraphPad Software, San Diego, CA, USA) was used for statistical analyses. Differences between groups were assessed by using the non-parametric Mann–Whitney test. Unpaired or paired Student’s *t*-test was used to compare means when appropriate. The non-parametrical Wilcoxon matched pairs test was used to compare two related samples. The Spearman’s rank correlation coefficient *r* was used to measure the relationship between paired data. Two-way ANOVA followed, if significant interaction, by a Bonferroni *post hoc* test was used when appropriate. For small samples (<10), a Kruskal–Wallis test was chosen. Differences with *p* < 0.05 were considered significant (*p < 0.05, **p < 0.01, and ***p < 0.001).

## Results

### HPLC Coupled with Fluorescence Detection: A Reliable Approach to Assay CA Secretion from Single Adrenal Slice Supernatant

**Figure 1:**
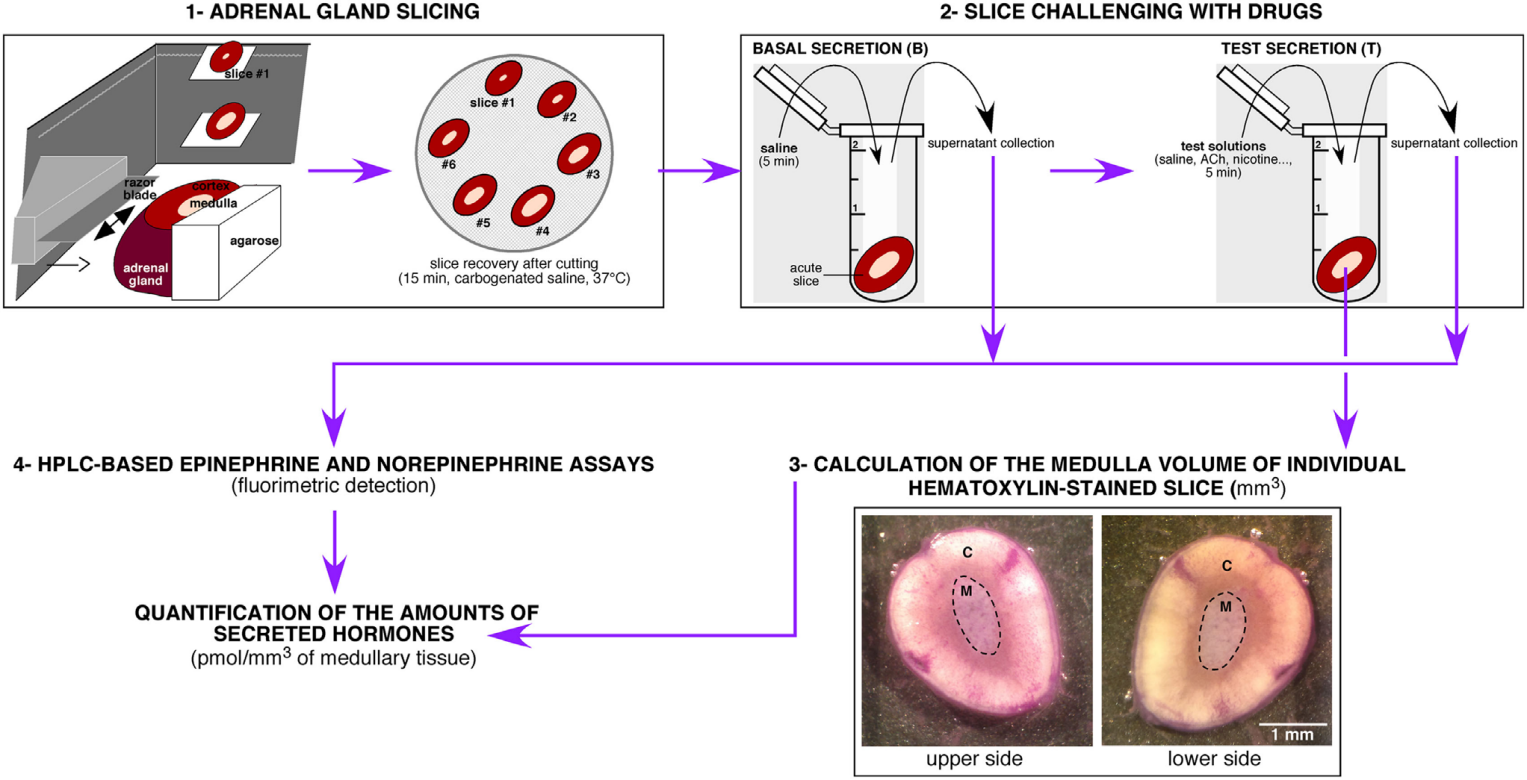
Schematic illustration of the experimental protocol leading to the high-performance liquid chromatography (HPLC)-based measurement of catecholamines [E and norepinephrine (NE)] in rat acute adrenal slice supernatants. The protocol can be divided into four main steps, including (i) the gland slicing using a vibratome, (ii) the challenge of individual slices with drugs of interest, (iii) the calculation of the medulla volume of each challenged slices after a hematoxylin staining, and (iv) the HPLC-based measurement of the amounts of E and NE released by each slice. C, cortex; M, medulla.

**Figure 2:**
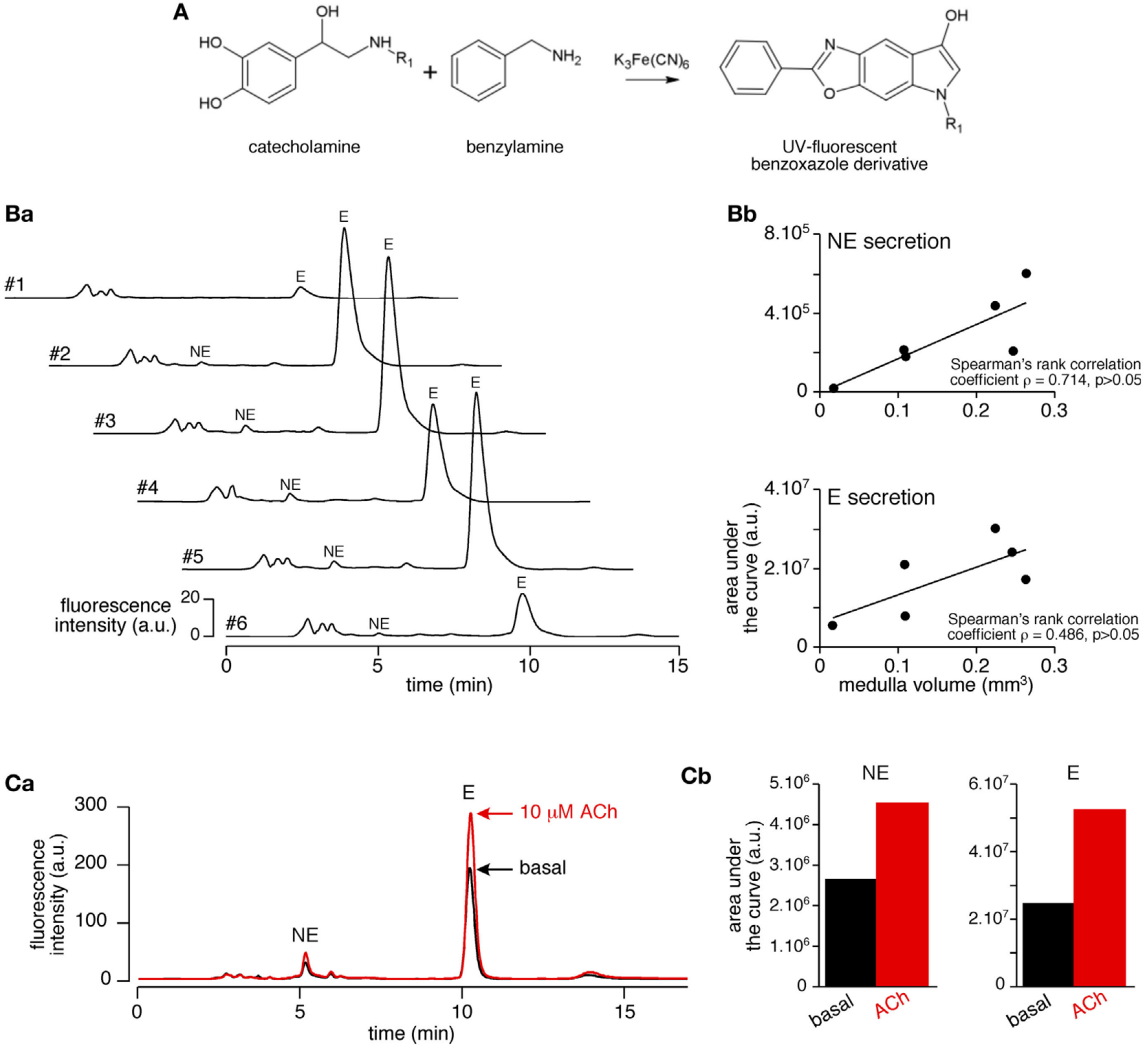
Validation of the use of high-performance liquid chromatography coupled with fluorescence detection for the determination of basal and stimulated catecholamine (CA) release in slice supernatants. **(A)** Chemical reaction leading to the production of the UV-fluorescent benzoxazole derivative. [**(B)**, a] Representative chromatograms of basal CAs released from six successive 150 μm-thick slices (#1 to #6, assay in 20 µl supernatant). [**(B)**, b] Plots of the correlation between hormone secretion [measured by the calculation of the area under the curve of E- and norepinephrine (NE)-related chromatographic peaks] and slice medulla volume {same slices than in panel [**(B)**, a]}. Although not statistically significant in the example illustrated in the figure, the Spearman’s rank correlation coefficient ρ indicates a positive correlation between the medulla volume and hormone chromatographic peaks. [**(C)**, a] Superimposed chromatograms illustrating the increased catecholamine (CA) (E and NE) content in a 10 µM acetylcholine chloride (Ach)-stimulated slice supernatant. [**(C)**, b] Calculation of the areas under the curve of NE- and E-related peaks allowing the quantification of CAs released before (basal) and after ACh stimulation.

A challenging issue of the study was to settle an experimental procedure allowing a reliable quantitative measurement of CA secreted by the medullary tissue from acute adrenal slices. As summarized in Figure 1, the protocol encompasses four main steps, that are (i) the tissue slicing, (ii) the slice challenging with drugs, (iii) the calculation of the adrenal medulla volume for each slice, and (iv) the HPLC-based quantification of the amounts of secreted CA (E and NE) per medulla unitary volume. Because the slicing procedure for the rat adrenal glands has been long described for both thick (>250 μm) and thin (150 µm) slices(3, 60), improvements of the experimental procedure mainly dealt with the HPLC-based CA assay. Contrasting with the routinely used electrochemical detection of CA, E and NE levels were determined here by a benzylamine-using pre-column derivatization followed by a fluorimetric detection (Figure 2A). For E and NE, LOD was 7 fmol for an injection volume of 20 µl. Representative chromatograms of basal E and NE secretion collected in 20 ml of supernatant of serial acute adrenal slices are shown in Figure 2B,a. Unlike NE, E was always reliably assayed, even from a small adrenal medulla volume (first and last slices). This observation is consistent with the fact that (i) the adult rat adrenal medulla harbors three to four times more E-secreting chromaffin cells than NE-secreting cells (61, 62) and (ii) the basal releasing rate for E is faster compared to NE (36). As expected, E- and NE-related chromatographic peaks calculated by measuring the area under the curve tend to positively correlate with the slice medulla volume (Figure 2B,b). The increased E and NE secretion in response to a cholinergic stimulation (10 µM ACh, Figure 2C) completed the validation of our experimental approach. Collectively, our results indicate that CA released from a single 150 μm-thickness adrenal slice can be successfully and reliably assayed, making use of HPLC with fluorescent detection-based method. This allows further valuation of the adrenal medulla secretory function, by considering new attributes such as left versus right gland and cellular localization near the cortex versus at the medulla center.

### Do the Left and the Right Adrenal Medullary Tissues Secrete Equal Amounts of E and NE?

**Figure 3:**
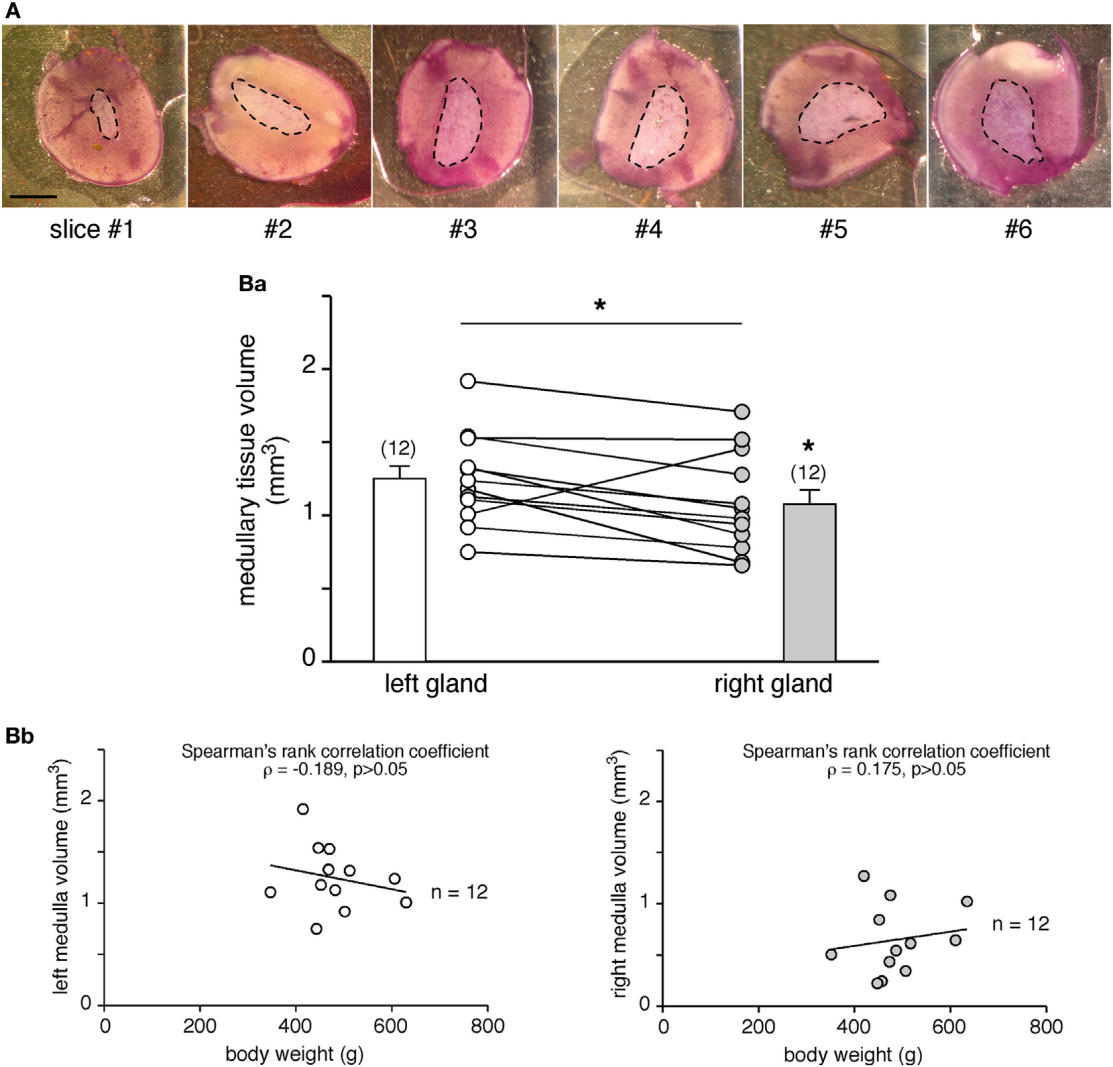
Comparison of the adrenal medulla volume between the left and the right glands. **(A)** Pictures illustrating the medulla surface (dashed line) in six serial slices (hematoxylin labeling) of the same adrenal gland. As expected from the anatomy of the rat adrenal gland and the central location of the medullary tissue, the medulla is smaller in the first cut slices (#1 and #2) and gradually increases in the deeper slices (#4, #5, and #6). Scale bar: 1 mm. [**(B)**, a] Calculation of the medulla volume in the left and right glands of 12 rats. The statistical analysis of the paired data indicates that the left medullary tissue is significantly greater than the right one (non-parametrical Wilcoxon’s matched pair test, *p < 0.05). [**(B)**, b] No significant correlation between the medullary tissue volume and the body weight (Spearman’s rank correlation coefficient ρ = −0.189 for the left gland, *p* > 0.05 and ρ = 0.175 for the right gland, *p* > 0.05).

The left and the right adrenal glands are not fully twins, but exhibit some distinct anatomical characteristics, which are especially marked for the innervation and vascularization tracks (54–56, 63). Because these distinguishable features may manage distinct secretory behaviors, we addressed the question of whether the two glands behave similarly in terms of amounts of hormones secreted. Taking advantage of our experimental procedure allowing quantification of the CA amounts secreted from a single adrenal slice, we tackled this issue by monitoring CA secretion in all slices from each gland and calculating the corresponding medullary tissue volume (Figure 3). The hematoxylin staining (Figure 3A) enables the estimation of the medulla surface of upper and lower sides of each slice, and then the volume calculation. As illustrated in Figure 3B,a, in adult rats, the medullary tissue volume averaged at ~1 mm^3^ and, interestingly, significantly differed between the left (1.25 ± 0.09 mm^3^, *n* = 12) and the right glands (1.08 ± 0.10 mm^3^, *n* = 12, *p* < 0.05, Wilcoxon matched pairs test), resulting in a mean right to left ratio of 0.88 ± 0.06 (*n* = 12). These data are consistent with the right:left ratio calculated for the whole adrenals [mean of 0.93 (64)]. However, no correlation was found when plotted over the body weight (Figure 3B,b).

**Figure 4:**
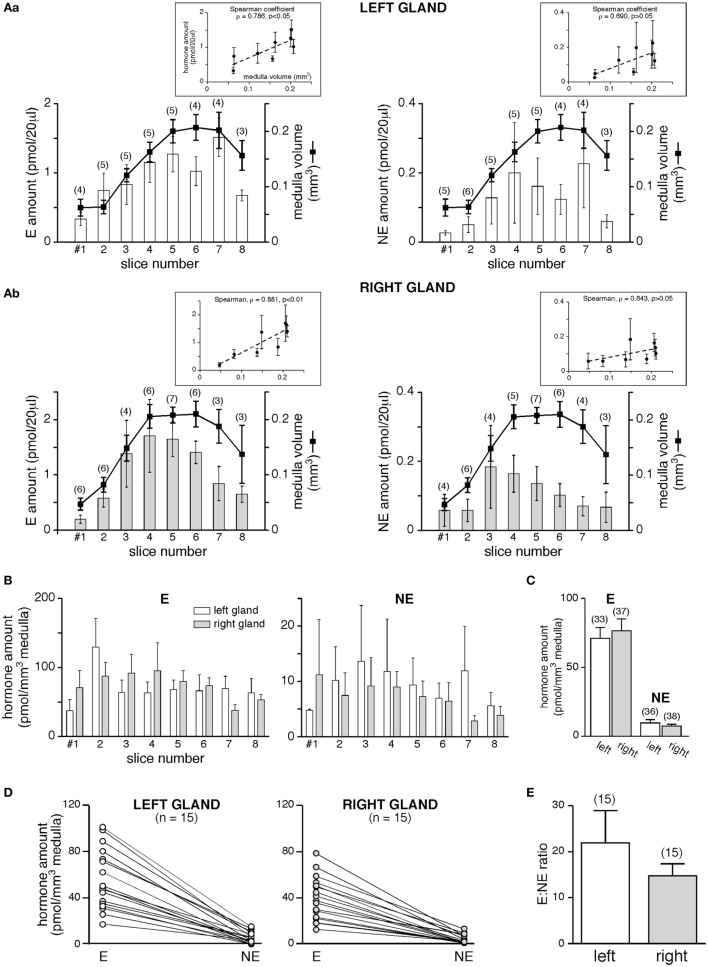
Basal catecholamine (CA) secretion from the left and the right gland. **(A)** CAs amounts [E, left panels and norepinephrine (NE), right panels] assayed in 20 µl supernatant collected from eight consecutive slices, and comparison to the medulla volume of associated slices. Pooled data from three to six left glands [**(A)**, a] and two to seven right glands [**(A)**, b]. The Spearman’s rank correlation coefficient ρ between the secreted hormone amount and the slice medulla volume is shown in insets. For E, larger is the medullary tissue volume in a slice, greater is the secreted hormone amount (ρ = 0.786 and 0.881 for the left and the right glands, respectively). For NE, no significant correlation is found between the secreted amount and the medulla volume. **(B–E)** E and NE are secreted equally by the left and the right glands. **(B)** Data expressed as a function of slice serial position [same slices as in panel **(A)**]. **(C)** Pooled data of all slices. **(D–E)** E:NE ratios calculated for the left and the right gland. Raw results in panel **(D)**, showing a greater secretion of E versus NE, for both the left and the right adrenal (each dot illustrates a single slice). **(E)** No significant difference in the E:NE ratio between the two glands.

Is the different medulla volume associated with different amounts of CA secreted by the two glands? We first addressed this issue on basal hormone release (Figure 4). The monitoring of basal E and NE secretion from individual slices clearly showed that for each hormones equal amounts are released from the left and the right gland (Figures 4A,B). However, when analyzing the secreted hormone amounts as a function of the medulla volume of each slice, a positive and statistically significant correlation was observed for E (Spearman’s rank correlation coefficient ρ = 0.786, *p* < 0.05 for the left gland and ρ = 0.881, *p* < 0.01 for the right gland, Figure 4A,a,b, left panels), but not for NE (ρ = 0.790, *p* > 0.05 and ρ = 0.643, *p* > 0.05, for the left and right glands, respectively, Figure 4A,a,b, right panels). This result likely reflects the lower proportion of NE-secreting chromaffin cells (~20%) versus E-secreting cells (~80%) within the rat medullary tissue (61, 62), as well a miscellaneous distribution of NE cell clusters within the medulla. Collective data (Figures 4B,C) show that all slices from either the left and the right glands display an equivalent secretory behavior, resulting in an equal amount of E and NE released per cubic meter medulla for the two adrenals (for E: 70.86 ± 8.05 pmol/mm^3^, *n* = 33 for the left gland versus 76.53 ± 8.61 pmol/mm^3^, *n* = 37 for the right gland; for NE: 9.78 ± 2.38 pmol/mm^3^, *n* = 36 for the left gland versus 7.43 ± 1.35 pmol/mm^3^, *n* = 38 for the right gland, *p* > 0.05, unpaired *t*-test). Whether for the left or the right adrenal, a single slice still secretes more E than NE (Figure 4D). Regarding this, it is noteworthy that the basal E:NE ratio did not significantly differ between the two glands (mean 22:1 for the left and 15:1 for the right adrenal; *p* > 0.05, Student’s *t*-test, Figure 4E).

**Figure 5:**
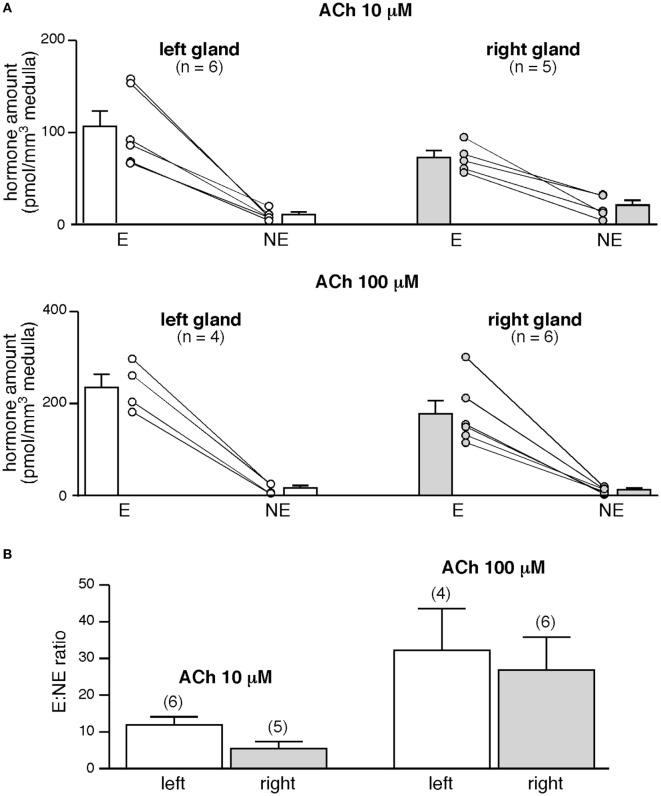
Acetylcholine-evoked catecholamine secretion from the left and the right glands. **(A)** Slices were challenged with a 5-min bath application of acetylcholine chloride (Ach) (10, upper graph or 100 µM, lower graph). Regardless of ACh concentration and of the left or right gland, E is main hormone secreted by individual slices. E:norepinephrine (NE) ratio is dose dependent but does not differ between the left and the right gland **(B)**.

To further investigate whether the left and the right glands release equal amounts of CA under stimulated conditions, the slices were challenged with a 5-min bath applied 10 or 100 µM ACh, the endogenous agonist of chromaffin cell cholinergic receptors (Figure 5). E and NE amounts secreted in response to ACh are dose-dependent and, for a given hormone, do not significantly differ between the two glands (*p* > 0.05, Mann–Whitney test, Figure 5A, white and gray bars). Unexpectedly and unlike what we previously observed for basal E and NE release (Figure 4C), the left adrenal has a propensity to secrete more CA than the right adrenal in response to an acetylcholine stimulation (for E: +45 and +21% for 10 and 100 mM ACh, respectively, and for NE: −52 and +42%, Figure 5A, white and gray bars). However, the stimulated E:NE ratio for the two ACh concentrations does not significantly differ between the two glands (Figure 5B), indicating that the left and the right glands exhibit an equivalent competence to release CA, at least in response to a cholinergic stimulation.

### Do the Periphery and the Center of the Chromaffin Tissue Secrete Equal Amounts of CAs?

**Figure 6:**
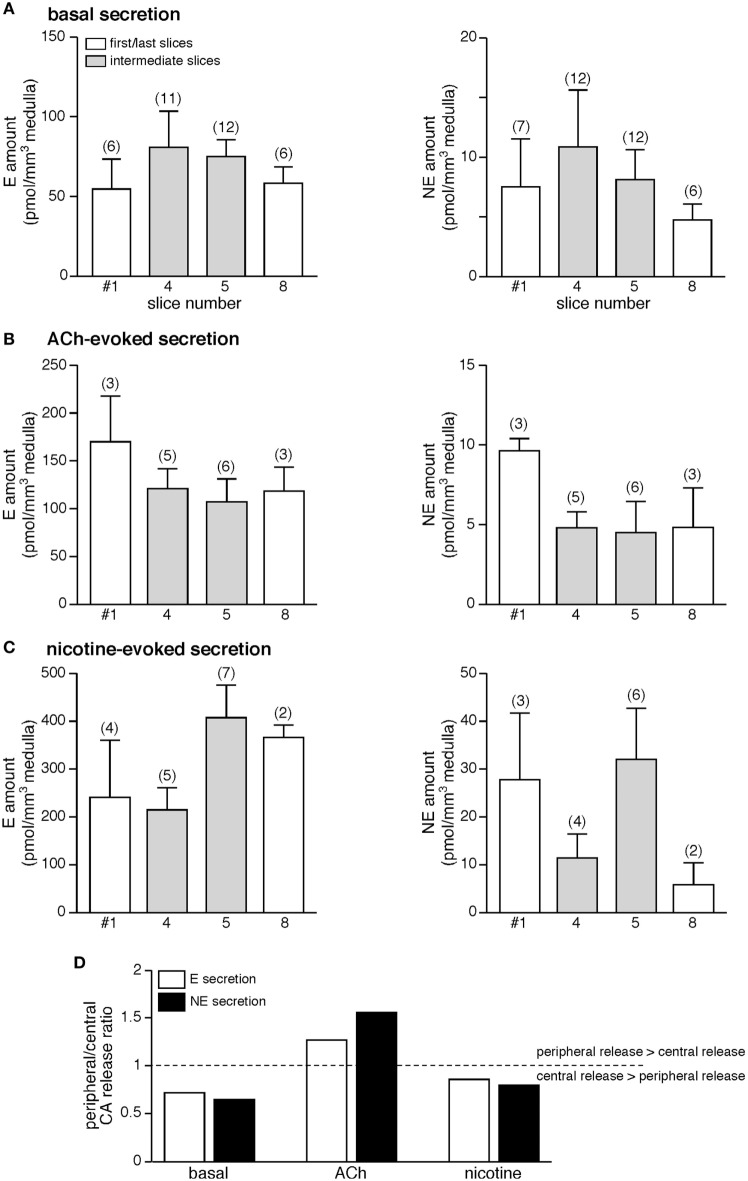
No meaningful difference in the amounts of hormone released from slices containing chromaffin cells mostly located close to the cortex or in the medulla center. Due to the round-shaped morphology of the adrenal glands, the first and last cut slices (white bars) contain numerous chromaffin cells that are in the vicinity of the cortical region. By contrast, the intermediate slices (gray bars) mostly contain chromaffin cells that are distant from the cortex. When comparing the amounts of E and norepinephrine (NE) released from first/last slices to hormones released from intermediate slices, no significant difference is observed, whether for basal secretion **(A)**, acetylcholine chloride (Ach)-evoked **(B)**, and nicotine-evoked secretion **(C)**. **(D)** Histogram illustrating the ratio peripheral/central catecholamines (CAs) release under basal, ACh-evoked, and nicotine-evoked hormone secretion. Note the bias toward a shift from central to peripheral E and NE release upon ACh challenges.

A remarkable feature of chromaffin cells located at the periphery of the medulla is their close vicinity to cortical cells and therefore their exposition to paracrine factors released by the cortex (57). Because this cortical-chromaffin cell cross talk has been described to impact chromaffin cell function, and reciprocally (57, 65), it was of interest to investigate whether or not a differential basal and/or stimulated CA secretion occurs depending on the chromaffin cell location (at the medulla edge or at the center). Serial slices were used to address this issue. We compared basal, ACh-evoked, and nicotine-evoked E and NE secretion of the first (#1) and the last (#8) slices (slices with a preponderance of chromaffin cells located close to the cortex) with secretion originated from intermediate (#4 and #5) slices (slices with a majority of chromaffin cells with a central position within the medulla). As illustrated in Figure 6A, the basal amounts of E (left panel) and NE (right panel) per cubic meter medulla do not statistically differ between first/last and intermediate slices (*p* > 0.05, Kruskal–Wallis test). A similar result was found for ACh-evoked (Figure 6B) and nicotine-evoked (Figure 6C) CA release. Although not statistically significant, the comparison between basal and stimulated CA secretion shows a bias toward a shift from central to peripheral E and NE release upon ACh challenges (Figure 6D). Collectively, our results indicate that, at least over the monitoring time period, whether chromaffin cells localize at the juxtamedullary region or reside in a more central position do not functionally impact the stimulus-secretion coupling, thus leading to an uniform basal or stimulated CA secretion throughout the chromaffin tissue. This, of course, does not exclude the occurrence of long-lasting modulation of the cortex on CA secretion.

### Comparison of CA Secretion upon an ACh or a Nicotine Challenge

**Figure 7:**
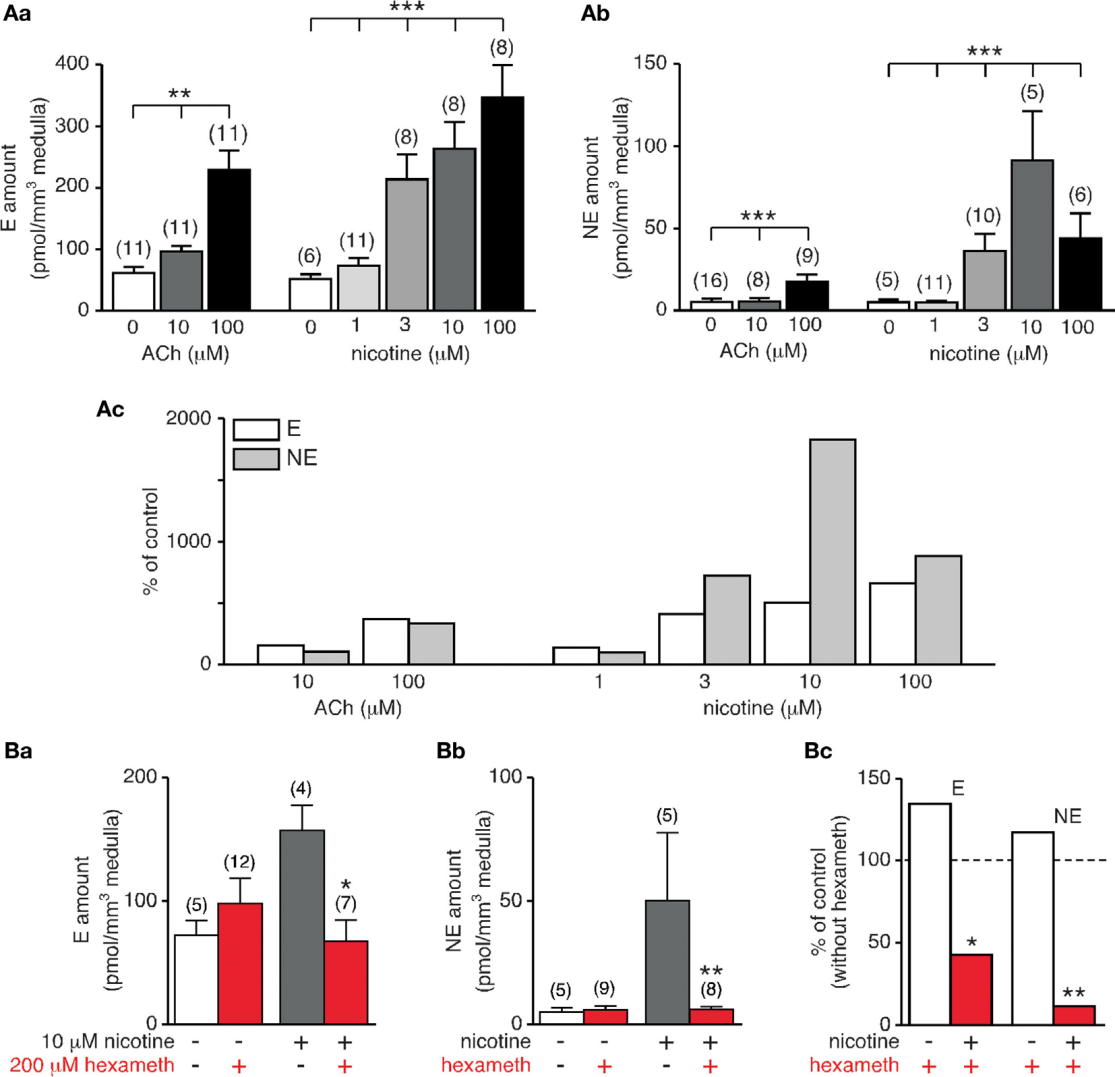
Dose-dependent acetylcholine chloride (Ach)- and nicotine-evoked catecholamine secretion and blockade by hexamethonium. **(A)** Individual slices were stimulated for 5 min by either ACh (10 and 100 µM) or nicotine (1, 3, 10, and 100 µM). Both E [**(A)**, a] and norepinephrine (NE) [**(A)**, b] release dose-dependently increases with agonist concentration. Note that for a same agonist concentration, nicotine is more efficient to evoke E and NE secretion [**(A)**, c]. **(B)** Effect of the α3-containing nAChR antagonist hexamethonium. Slices were incubated for 5 min with hexamethonium (200 µM) before challenging with 10 µM nicotine (or saline for control experiments). While basal E and NE secretion is not affected by hexamethonium, nicotine-triggered E [**(B)**, a] and NE [**(B)**, b] release is significantly reduced. [**(B)**, c] Pooled data illustrating a strongest ability for hexamethonium to reduce NE secretion compared to E secretion (88 versus 57% decrease). *p < 0.05, **p < 0.01, ***p < 0.001.

Because CA secretion in rat is chiefly evoked by stimulation of postsynaptic nAChRs, we monitored E and NE secretion in response to increasing concentration of nicotine (1, 3, 10, and 100 µM) and we compared the CA amounts released to that evoked upon an ACh stimulation (10 and 100 µM). As expected, E and NE were dose-dependently secreted (*p* < 0.05, Kruskal–Wallis test, Figure 7A,a,b). Comparing the amounts of E and NE released for a same agonist concentration shows that nicotine is more efficient that ACh to induce hormone secretion. In addition, differential effects on E and NE secretion were observed in response to the nicotinic challenge (Figure 7A,c). While the percentage of stimulation remains comparable for E at 3, 10, and 100 µM nicotine, (i) the stimulation efficiency for NE appears less homogeneous with an obvious difference at 10 µM nicotine and (ii) nicotine, but not ACh, appears more efficient to evoke NE secretion that E secretion.

In rat adrenal chromaffin cells, nAChRs are prevalently built by the α3 subunit, associated with β4 or β2 subunits with the addition of the ancillary α5 subunit (66). Moreover, α3 subunit-containing nAChRs are the major subtype transducing the ACh-evoked postsynaptic electrical response in chromaffin cells (67). We therefore next examined the effects of hexamethonium, an uncompetitive and selective nAChR antagonist of α3-containing receptors (68), on both basal and nicotine (10 μM)-evoked CA secretion (Figure 7B). As shown in Figure 7B,a–c, hexamethonium (200 µM) does not significantly affect basal E and NE release (*p* > 0.05), but drastically reduce nicotine-stimulated E and NE secretion, although in a different extent (57 and 88% decrease for E and NE secretion, respectively). The highest sensitivity of NE secretion to hexamethonium suggests a differential expression of nAChRs subtypes between E- and NE-secreting chromaffin cells, with a more robust expression of α3-containing receptors in NE cells.

## Discussion

In this study, we report the use of HPLC coupled with fluorescence detection as a sensitive and easy method to reliably monitor basal and stimulated CA (E and NE) amounts in rat adrenal slice supernatants. Unlike countless CA assays performed from the circulating blood, this method combining slice supernatant collection to a quantitative detection of hormone secretion by HPLC provides a monitoring of CA amounts directly secreted by the adrenal chromaffin tissue and enables comparisons between the right and the left glands. This experimental approach is also well adapted for pharmacological experiments, as several drugs can be preliminary screened in parallel in many slices. In addition, the fluorescence detection displays several advantages. First, the detection limit (for a signal-to-noise ratio of 3), compared to electrochemistry-based assays and gas chromatography/mass spectrometry (MS) and liquid chromatography/MS (20, 69), is greater, with measurements in the attomole range (69–73). Second, and unlike the electrochemical detection, the sample nature (plasma, urines, tissue homogenates, etc.) does not impinge on the assays. Indeed, the sensitivity and quality of the quantification by electrochemical detection can be affected by the oxidation of the catechol groups and by possible chromatographic interferences with coeluted compounds (74, 75). Third, the benzylamine derivatization of CA stabilizes the samples at room temperature and therefore advantages their short-term storage before separation and detection (33). CA derivatization with benzylamine requires a unique pre-column sample derivatization without any other treatment before injection into the HPLC column, and is therefore a very easy method to implement. In addition and as shown in this study, fluorescence-based CA detection is a suitable method to assay both E and NE from adrenal slice supernatants. The method is therefore (i) appropriate to measure E and NE in a saline solution, (ii) sensitive enough to monitor E and NE in small volume samples [detection limit of 7 fmol for 20 µl injected, consonant with previous studies (33, 76)], and (iii) fast since the two biogenic amines are eluted within 10 min. As such, the fluorescent-based CA assay using a pre-column derivatization is an easy-to-use technique combining sensitivity and speed and is therefore a method quite convenient for routine analysis. It is noteworthy that biological fluids (plasma and urines), unlike slice supernatants, contain numerous compounds, which can impact CA detection. In this case, MS detection is a good alternative (18, 20, 77). In particular, the technical approach using HPLC/MS/MS can allow the quantification of a wide panel of secreted components, opening thus perspectives to characterize the adrenal secretome in physiological and pathological conditions.

### Features of the Rat Adrenal CA Release: Lessons from the Left and Right Gland Secretory Behavior

The left and the right glands are not wholly twins and many distinctive clues can be found regarding their innervation and vascularization (54–56, 63, 78). We took advantage of our experimental approach to first refine the medullary volume for the two glands. Our calculated volume of ~1 mm^3^ coincides with the data reported by Tomlinson and colleagues in adult male Wistar rats (62). While it is well known that the left adrenal is greater than the right adrenal (79), with a mean right/left weight ratio of 0.93 (64), our results report for the first time that the adrenal medullary volume is also dimorphic, with a mean right/left volume ratio of 0.88. Does this dimorphism impact the secretory behavior of the adrenal? Unlike E, basal NE secretion monitored in serial slices does not reliably echo the medulla volume of each slice, both for the left and the right glands. This observation is consistent with the fact that NE-secreting cells represents only 20% of chromaffin cells in the rat (61, 62) and confirms that rat NE cells are irregularly distributed throughout the gland with no spatial preference (80).

Our experiments performed in parallel in the left and right glands allowed to estimate the E:NE ratio for the two glands. The basal ratio E/NE does not significantly differ between the left and the right adrenals. To the best our knowledge, this study is the first determination of the E:NE ratio for the right gland, less accessible than the left one for hormone collection. The ratio values that we calculated for the left gland are fully consistent with the values previously reported in the adrenal venous blood of anesthetized rats, ranging from 2 to 12 (37, 38, 81, 82). Interestingly, and coinciding with a previous report (83), E:NE ratio does not change after cholinergic (ACh or nicotine) stimulation. Also, the values are comparable to those measured *in vivo*, in response to stress or to nerve stimulation, in the adrenal venous blood (6, 36, 82) or in microdialyzed glands (37, 38).

### Additional Features of the Rat Adrenal CA Release: Lessons from E and NE Secretion

Being surrounded by the adrenal cortex, the medullary tissue strategically locates within the adrenal gland. In particular, whether the location of chromaffin cells at the periphery (i.e., close to the zona reticularis cells) or at the center of the medulla impacts E and NE release is a key, but laborious question to address. Our CA release experiments differentially performed in first/last slices (containing many chromaffin cells juxtaposed to cortical cells) versus intermediate slices (containing both peripheral and central chromaffin cells) did not disclose unequivocal changes in the amounts of secreted E and NE. This confirms that, unlike in the hamster medullary tissue in which NE-secreting cells preferentially position at the periphery of the chromaffin tissue (84), rat NE and E cells are not segregated in distinct areas throughout the medulla (80). Regarding E and NE secretion, we observed a bias toward a shift from central to peripheral E and NE release upon ACh challenges, suggesting that peripheral and central CA secretion might be differentially regulated. Along the same line, an attempt to answer this thorny issue has been recently reported using an elegant *ex vivo* spinal–splanchnic nerve–adrenal preparation (85). The authors showed that upon robust splanchnic nerve stimulation, E seems to be preferentially released from the periphery and NE secreted from the center of the medulla. This intriguing finding was, however, not statistically supported, rendering thus difficult the statement of an unambiguous conclusion. But it opens the way to a novel challenging issue.

Another interesting question that would deserve to be addressed concerns the kinetics of E and NE release. We did not investigate this point in the present study, because (i) our slice preparation does not integrate the blood outflow rate and (ii) others methodological approaches are much more appropriate to assess this issue. Indeed, amperometry and/or fast-scan voltammetry techniques allow monitoring CA secretion with milliseconds or seconds time resolution, as reported in superfused mouse adrenal slices (50). Note that previous *in vivo* studies (CA collected from the adrenal venous blood) have estimated the basal secretory rate for E at about 25–27 ng/kg/min, a rate seven to eight times faster than for NE (36, 81).

### Distinct Involvement of α3-Containing nAChRs in E and NE Secretion

Beyond the fact that our experimental approach combining adrenal slice supernatant collection and HPLC-based CA assays is well appropriate for pharmacological experiments, our study points to two novel findings of the role of α3-containing nAChRs in E and NE secretion. First, while substantially engaged in the secretory response evoked by a cholinergic stimulation, α3 nAChRs appear to not significantly contribute to basal CA release. This suggests the recruitment of other nAChRs. Homopentameric α7, α9, or α9/α10 nAChRs could be good candidates, since (i) they are expressed at the rat splanchnic nerve-chromaffin cell synapses and their co-activation contributes to spontaneous postsynaptic excitatory events (60, 86) and (ii) they exhibit a robust Ca^2+^ permeability (87, 88) compared with α3-containing nAChRs (89). Because an increase in cytosolic Ca^2+^ is a prerequisite for chromaffin cell exocytosis (90), it is likely that α7, α9, and/or α9/α10 nAChR activation by ACh would trigger instructive signaling cascades contributing to CA release from chromaffin cells.

Second, α3-containing nAChRs seem to be distinctly involved in E and NE secretion. Indeed, but with the limitation of the use of a single and high concentration of antagonist, hexamethonium is more efficient to reduce NE release than E release in response to a nicotinic stimulation. This finding is, however, consistent with studies showing that (i) nAChRs are primarily present on NE cells while both nAChRs and muscarinic receptors (mAChRs) are expressed in E cells (38) and (ii) E and NE-secreting chromaffin cells are differentially innervated by cholinergic inputs, NE cells receiving more ACh-containing fibers than E cells (91). In addition, a similar result has been described both in response to splanchnic nerve stimulations (38) and in response to ACh-evoked CA secretion in the presence of mecamylamine, a nAChR antagonist preferentially acting at αβ-containing nAChRs. More importantly, our results are also indicative of the expression of distinct nAChR subtypes in E and NE cells, pointing to a dominant expression of α3-containing nAChRs in NE-releasing cells. To date, an exhaustive description of the nAChR subtypes expressed in individual E- and NE-secreting rat chromaffin cells is missing. Also, the respective contribution of nAChR subtypes to rat CA secretion remains an open question.

### Distinct Involvement of nAChRs and mAChRs in E and NE Secretion

The comparison between ACh- and nicotine-induced CA release shows that nicotine more potently evokes NE exocytosis than ACh does, consistent with the observation that nAChRs are primarily expressed in NE-containing cells (38). Unlike nicotine that stimulates nAChRs only, ACh binds to both nAChRs and mAChRs. Taking these two cholinergic receptors into account puzzles a little more the situation. Indeed, activation of mAChRs can lead to opposite effects on CA secretion. By binding at M1 receptors, muscarinic agonists enhance chromaffin cell excitability (92, 93) and subsequent CA release (94). In addition, mAChRs can negatively regulate nAChRs, leading to decreased nicotine-evoked CA secretion (95).

### Possible Outlooks

Because easy to implement, the experimental approach described in this study opens a wide panel of possible applications, both in physiology, physiopathology, pharmacology or biochemistry. Interestingly, our experimental approach provides the study of the adrenal medulla, both at the chromaffin cell level (including the possibility to differentiate between E- and NE-releasing cells) and at the tissue level by independently monitoring the left and the right gland secretory behavior. Such a refinement of the adrenal CA secretion could be pertinent in many physiological (acute stress, aging, etc.) or pathological (chronic stress, stress-related endocrine diseases such as arterial hypertension, diabetes, obesity, and chromaffin cell tumors) conditions. From a pharmacological/toxicological point of view, the use of selective agonists or antagonists targeting nAChRs (or subtypes) or muscarinic receptors could help to better understand their respective contribution in CA secretion.

Along the same line, the effects of other agonists/antagonists potentially active on CA secretion could be investigated. Several biologically active factors (adenosine 5′ triphosphate, vasoactive intestinal peptide, substance P, neurotensin, enkephalin, galanin, chromogranins, etc.) are co-stored and co-secreted with CA, and can therefore facilitate or inhibit CA release, through positive or negative autocrine/paracrine feedback loops (96). CA by activating chromaffin cell beta-adrenoceptors can also impact their own release (97). The HPLC-based CA assay described in this study could provide a suitable methodological approach to address the respective contribution of each factor in the regulation of CA secretion by the adrenal medulla. Although not performed here, the fluorescent detection of other adrenal hormones such as corticosteroids, using a pre-column derivatization, is likely achievable (98–100). In addition, the application fields are not restricted to Endocrinology. Neurological pathologies affecting adrenal CA homeostasis [such as epilepsy (101, 102) or neurodegenerative diseases (103)] could also be targeted by this experimental approach.

As such, the HPLC-based quantitative analysis of CA from adrenal slice supernatant is an easy to implement method that provides robust and relevant physiological information on the adrenal secretory behavior and therefore represents an elegant strategy to complement both *in vitro* (cultured chromaffin cells) and *in vivo* (anesthetized animals) studies.

## Ethics Statement

All procedures in this study conformed to the animal welfare guidelines of the European Community and were approved by the French Ministry of Agriculture (authorizations no. 49-2011-18 and 49-247).

## Author Contributions

NG and CLegros conceived and designed the experiments. FN, CLefort, CLegros, and NG performed the experiments. DB and PR assisted with HPLC equipment. FN, CLegros, and NG analyzed the data. NG prepared the figures and wrote the paper with input from FN and CLegros.

## Conflict of Interest Statement

The authors declare that the research was conducted in the absence of any commercial or financial relationships that could be construed as a potential conflict of interest. The reviewer NV and handling editor declared their shared affiliation.
